# Supplementary figures and images for: Antimicrobials from a feline commensal bacterium inhibit skin infection by drug-resistant S. pseudintermedius
Source: eLife. 2021 Oct 19;10:e66793. doi: 10.7554/eLife.66793 (PMC8592530; doi:10.7554/eLife.66793)

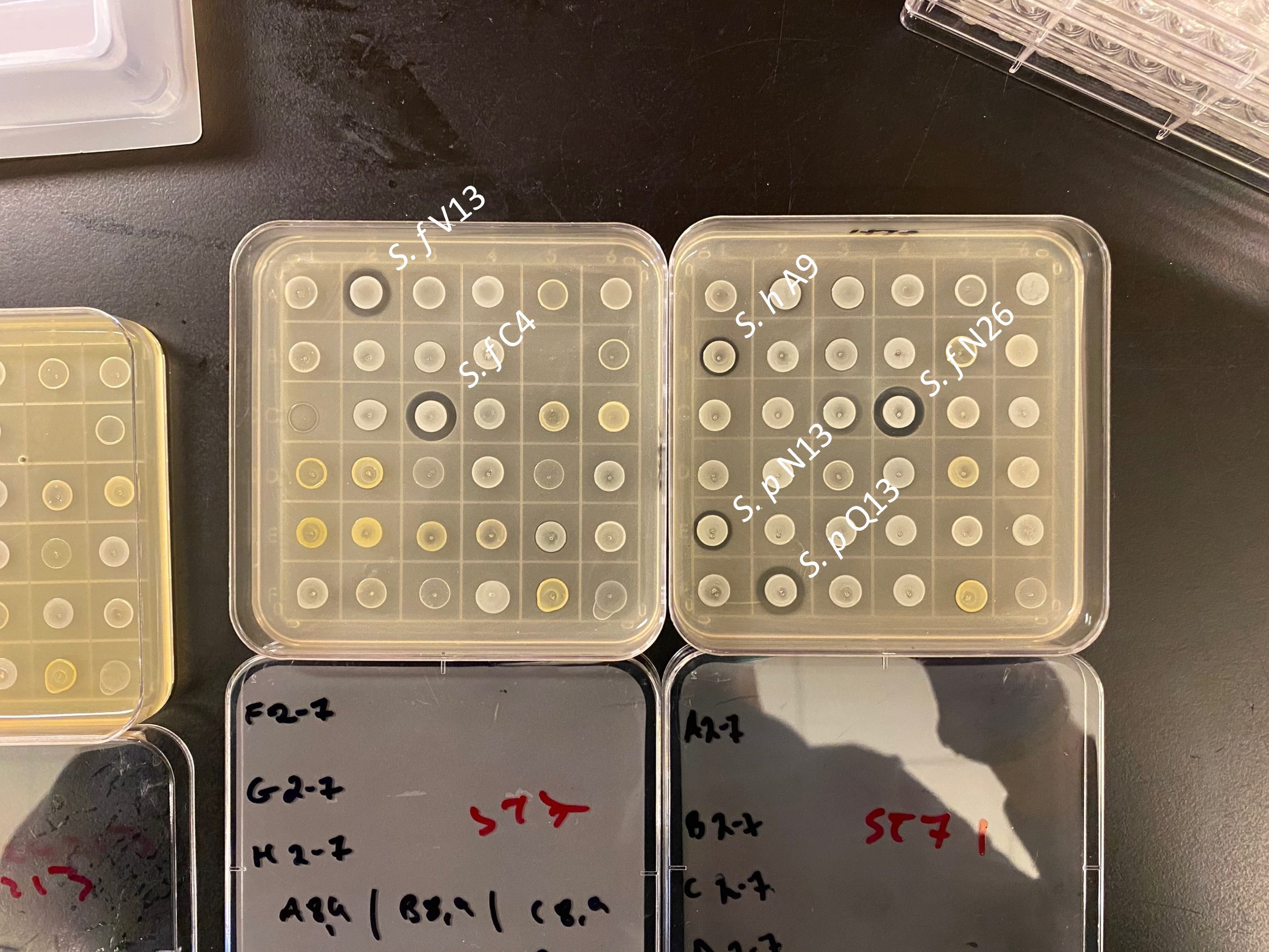

Supplement: Figure 1—source data 2. [file elife-66793-fig1-data2.zip › Figure 1-labelled source data 2.tif]

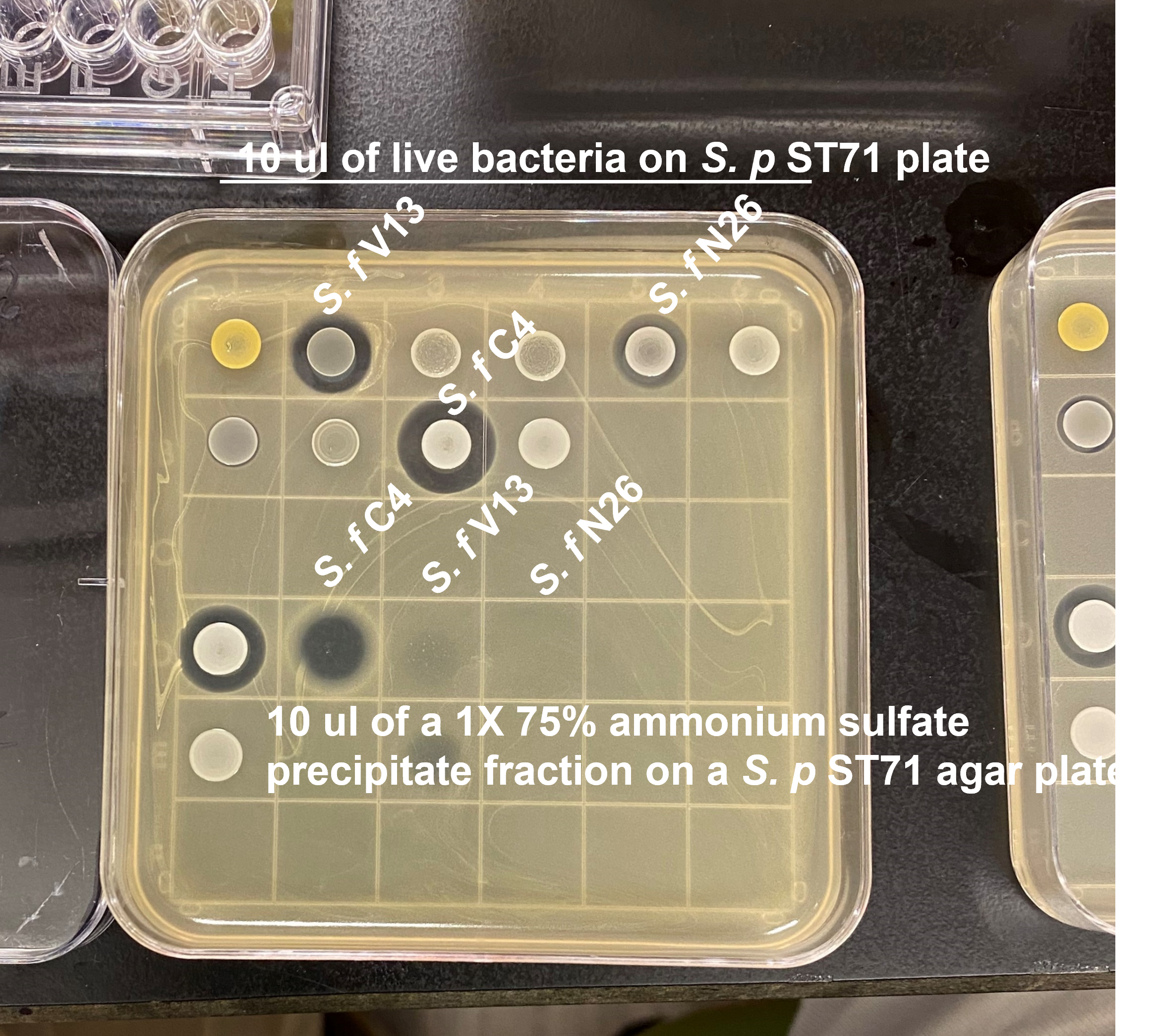

Supplement: Figure 1—figure supplement 1—source data 1. [file elife-66793-fig1-figsupp1-data1.zip › Figure 1-figure supplement 1 labelled source data 1.tif]

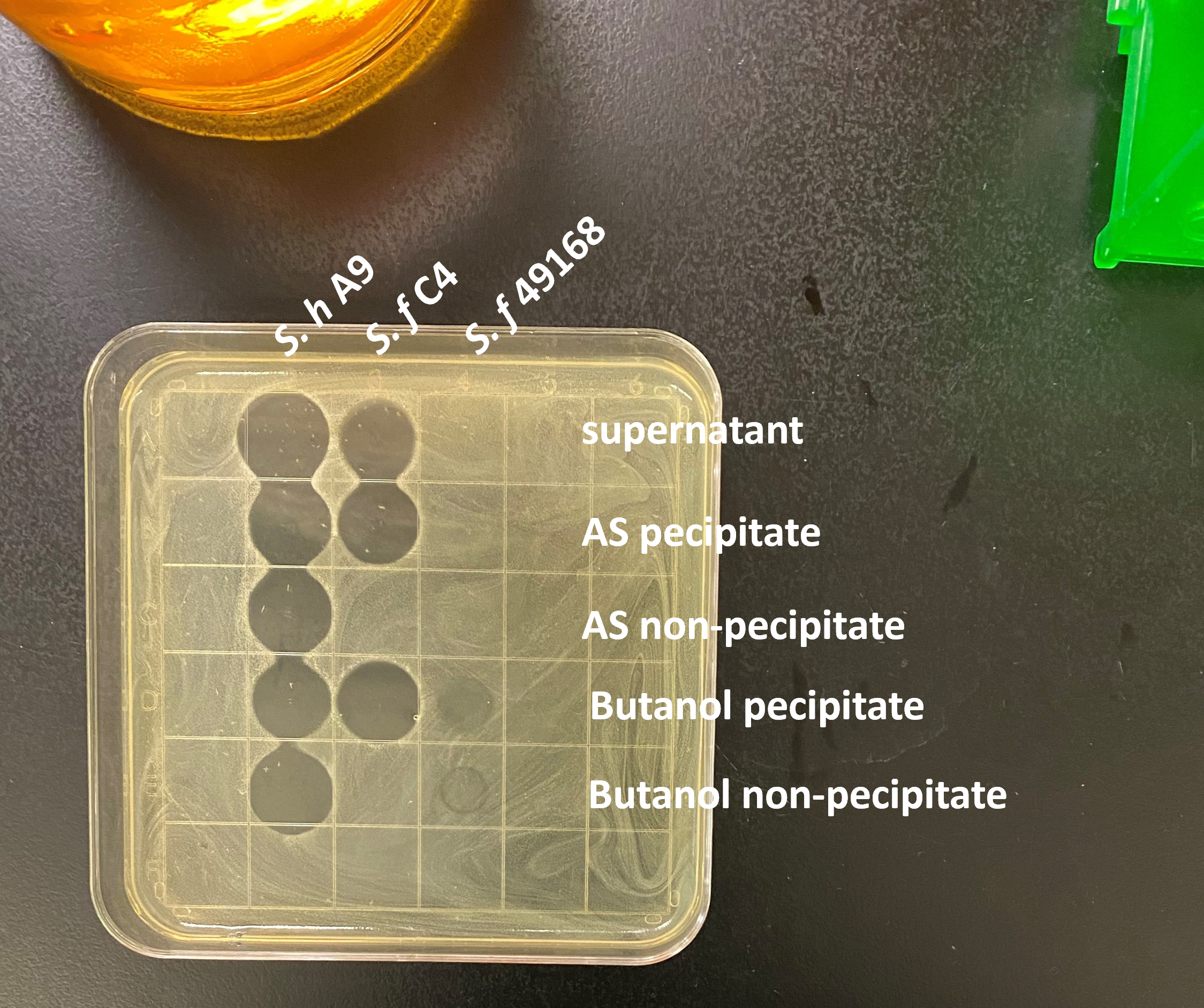

Supplement: Figure 1—figure supplement 1—source data 2. [file elife-66793-fig1-figsupp1-data2.zip › Figure 1-figure supplement 1 labelled source data 2.tif]

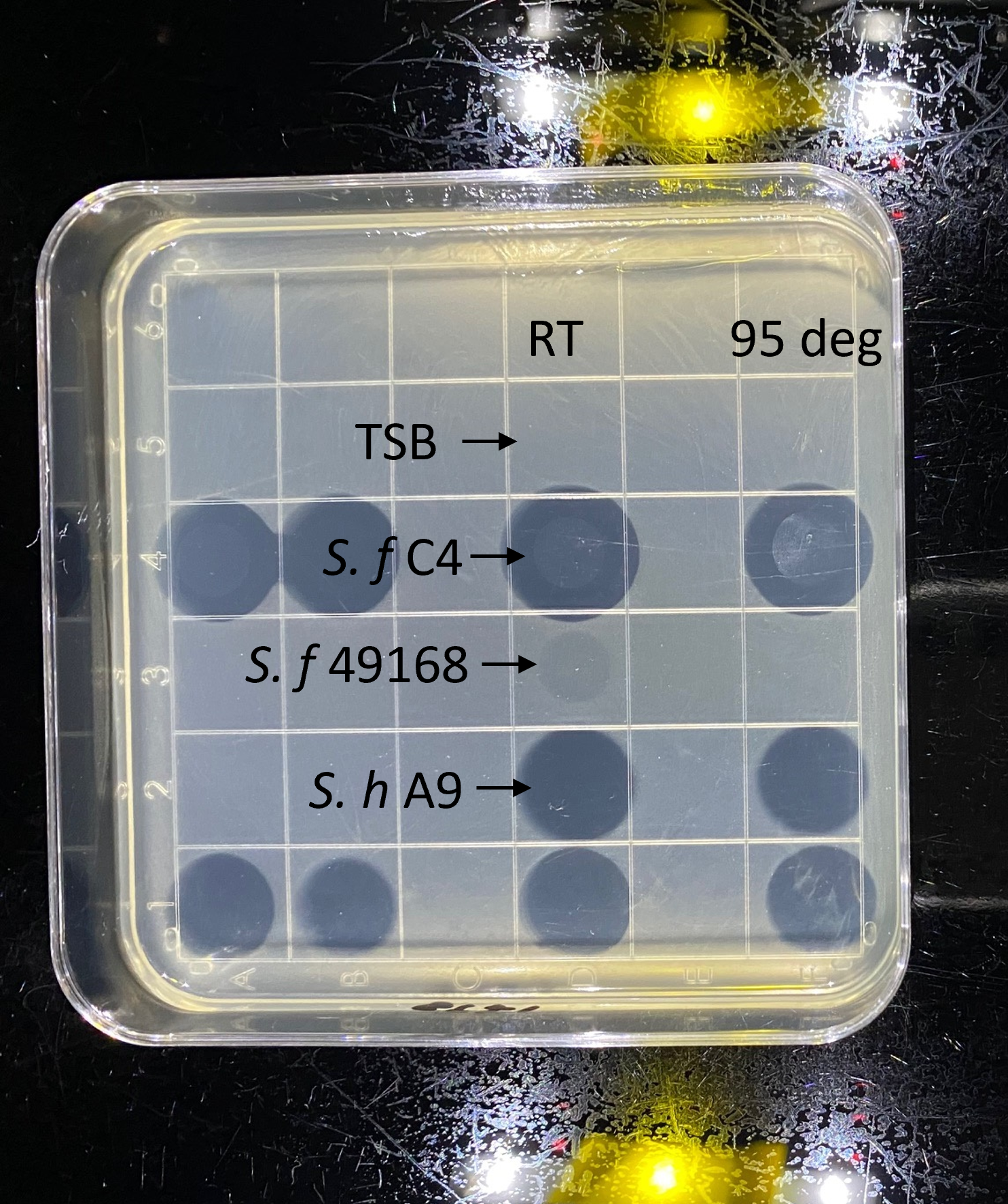

Supplement: Figure 1—figure supplement 1—source data 3. [file elife-66793-fig1-figsupp1-data3.zip › Figure 1-figure supplement 1 labelled source data 3.tif]

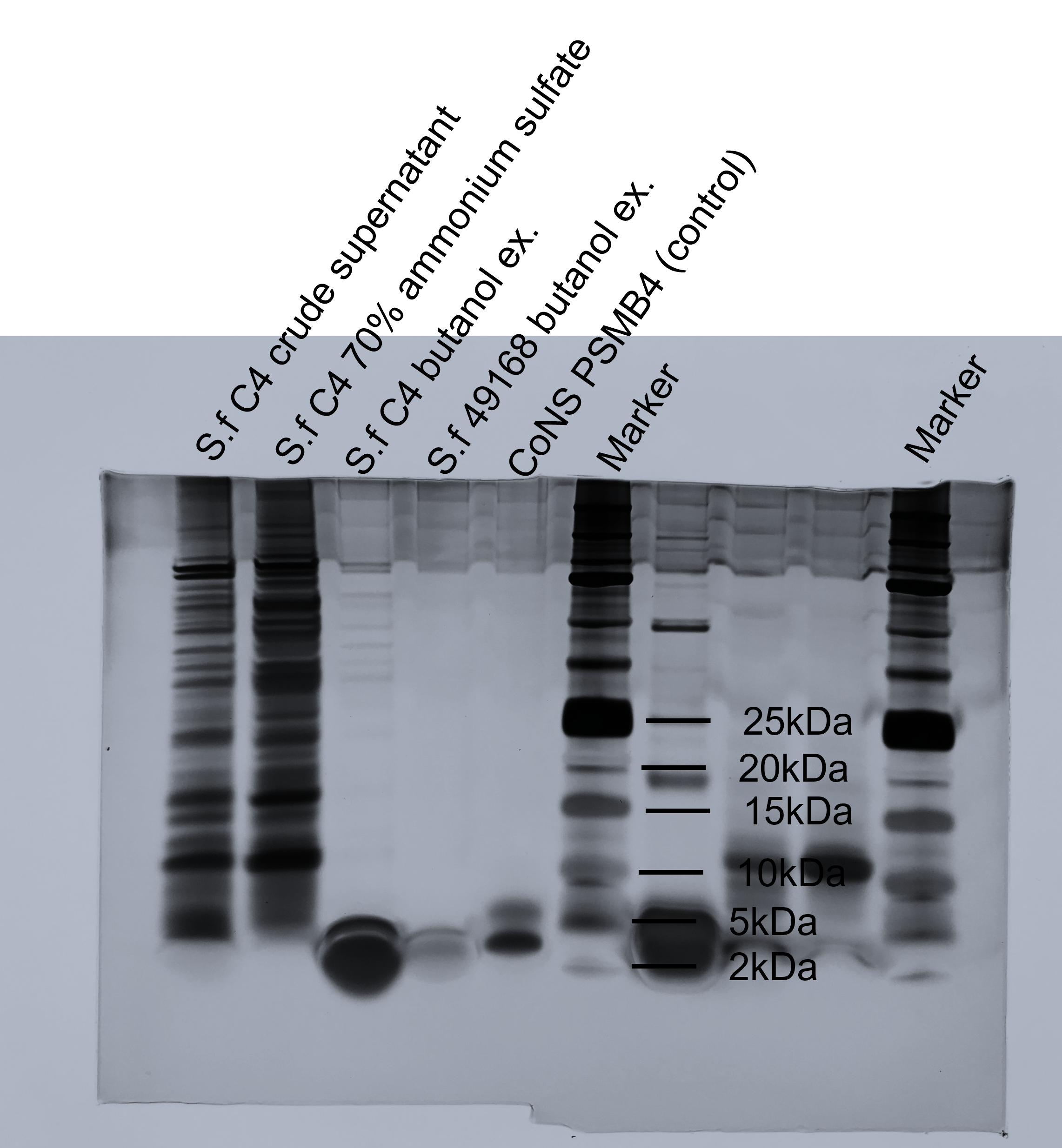

Supplement: Figure 1—figure supplement 1—source data 4. [file elife-66793-fig1-figsupp1-data4.zip › Figure 1-figure supplement 1 labelled source data 4.tif]

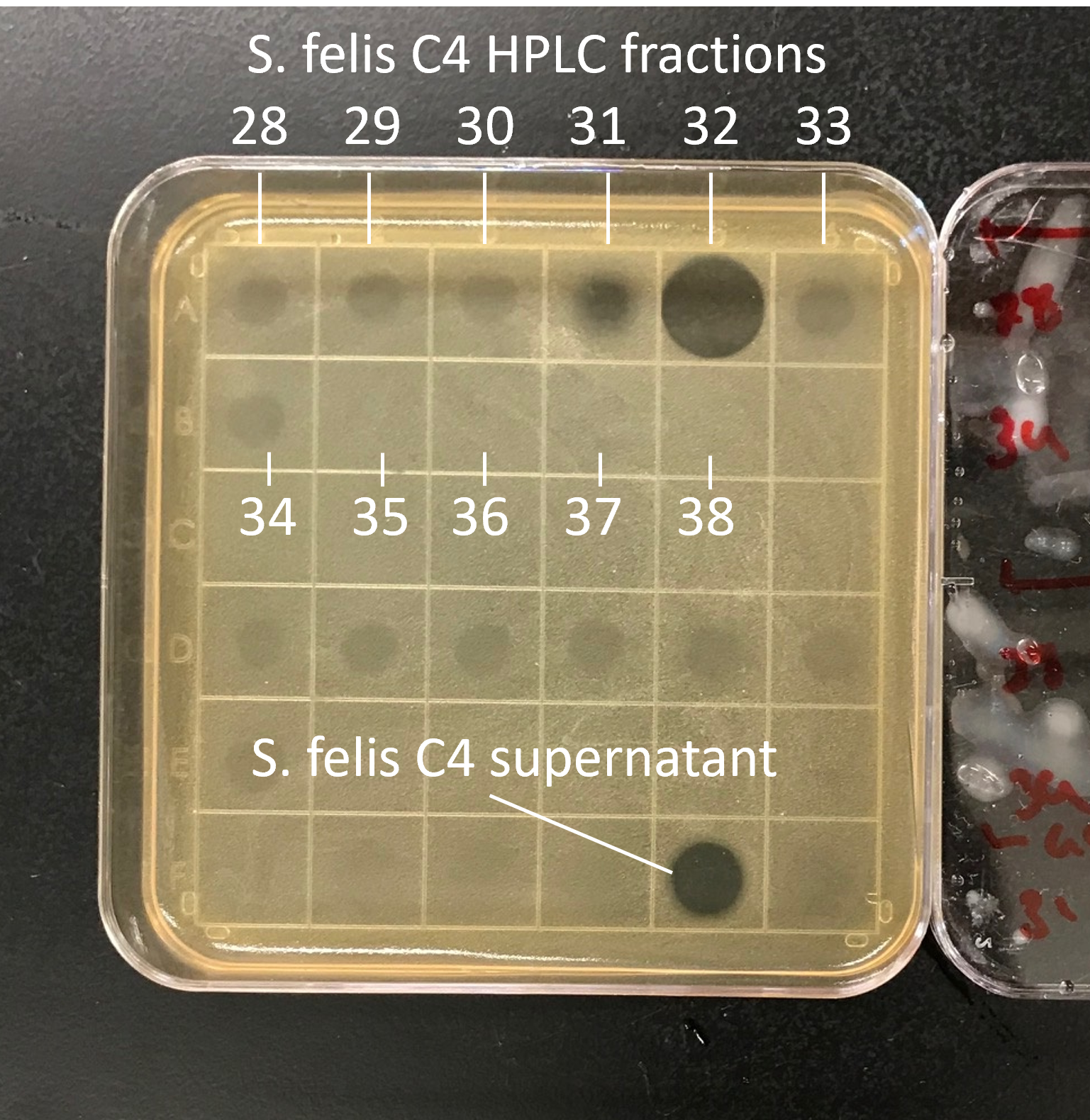

Supplement: Figure 2—source data 1. [file elife-66793-fig2-data1.tif]

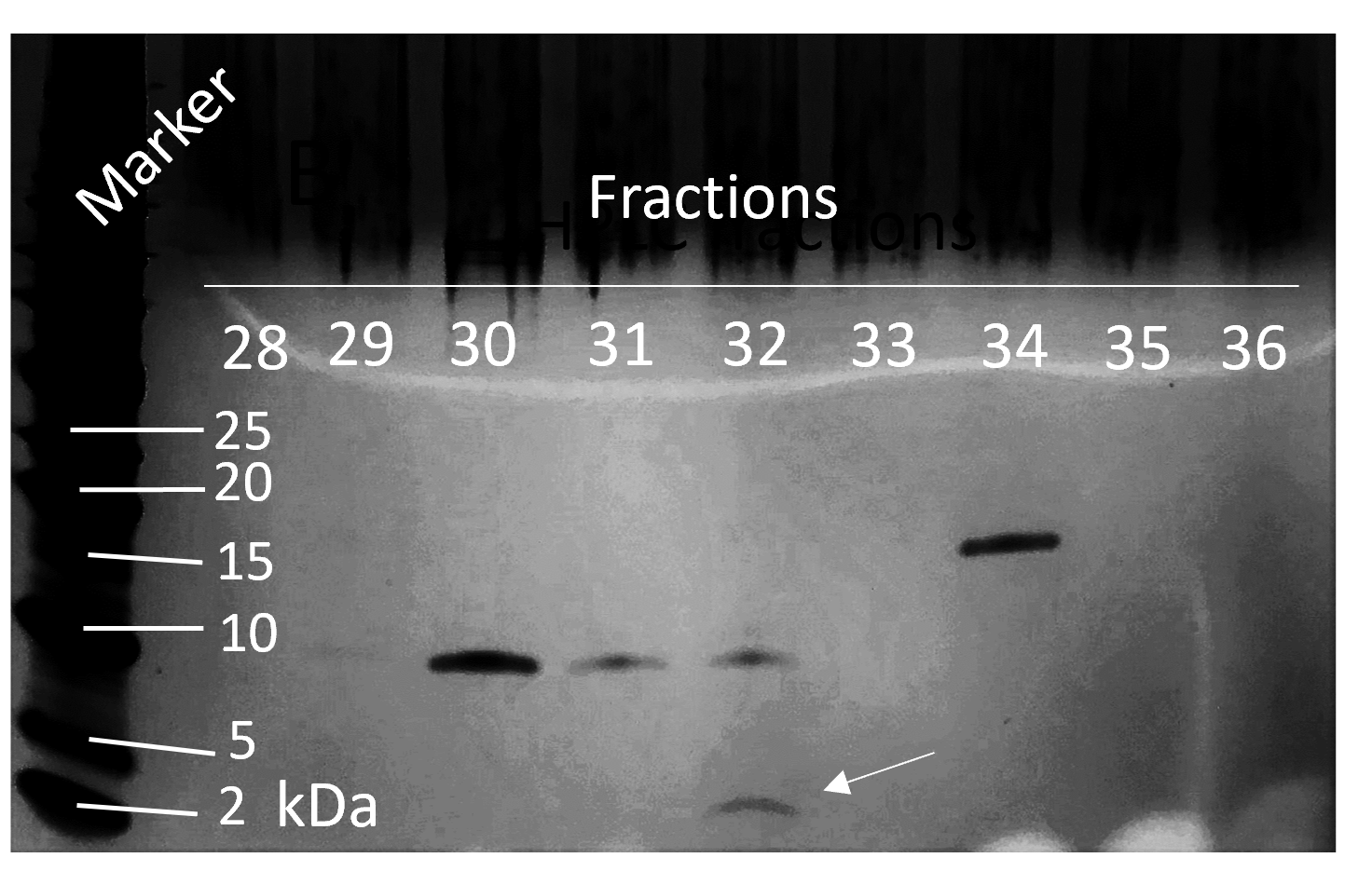

Supplement: Figure 2—source data 2. [file elife-66793-fig2-data2.tif]

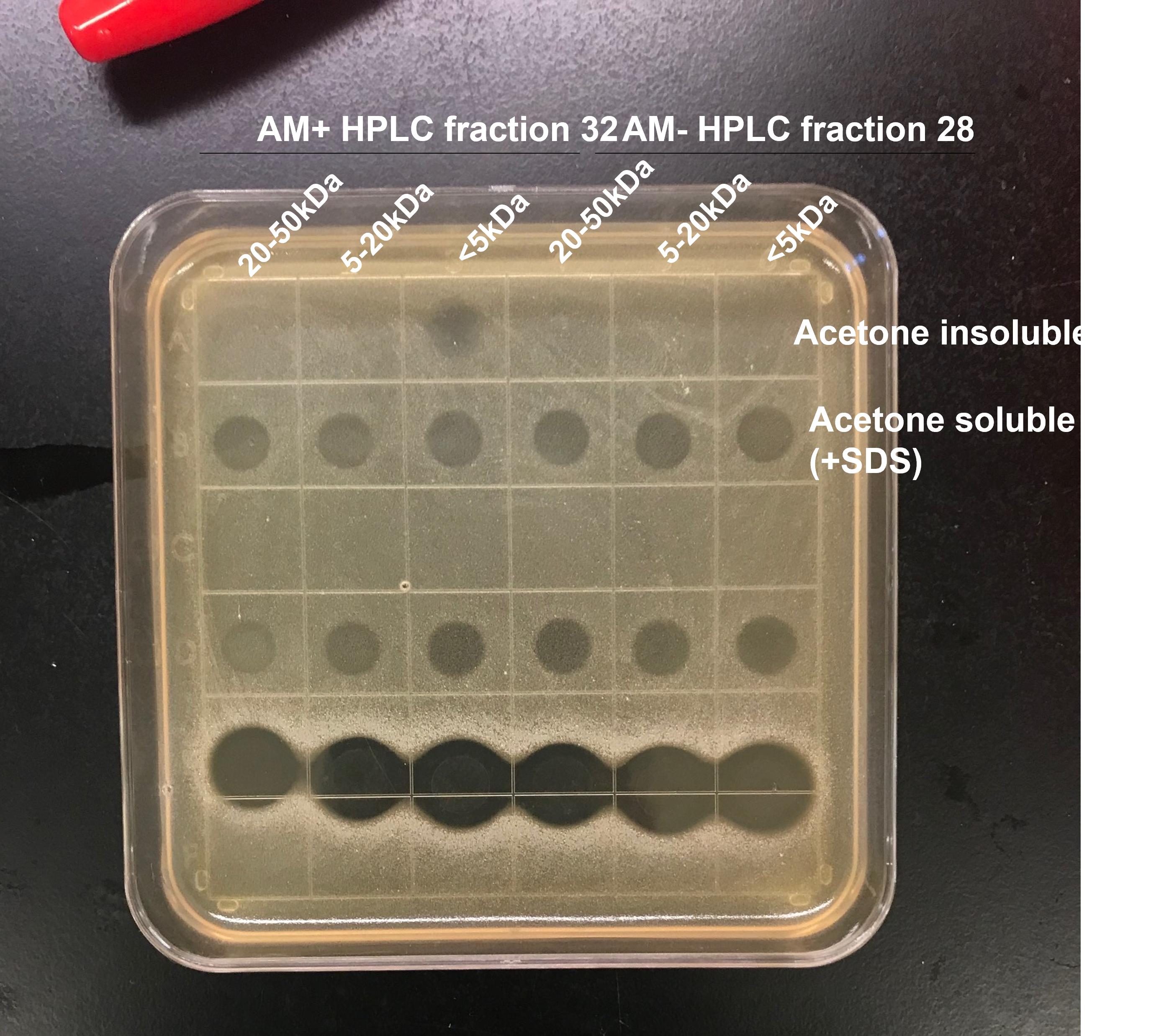

Supplement: Figure 2—source data 3. [file elife-66793-fig2-data3.tif]

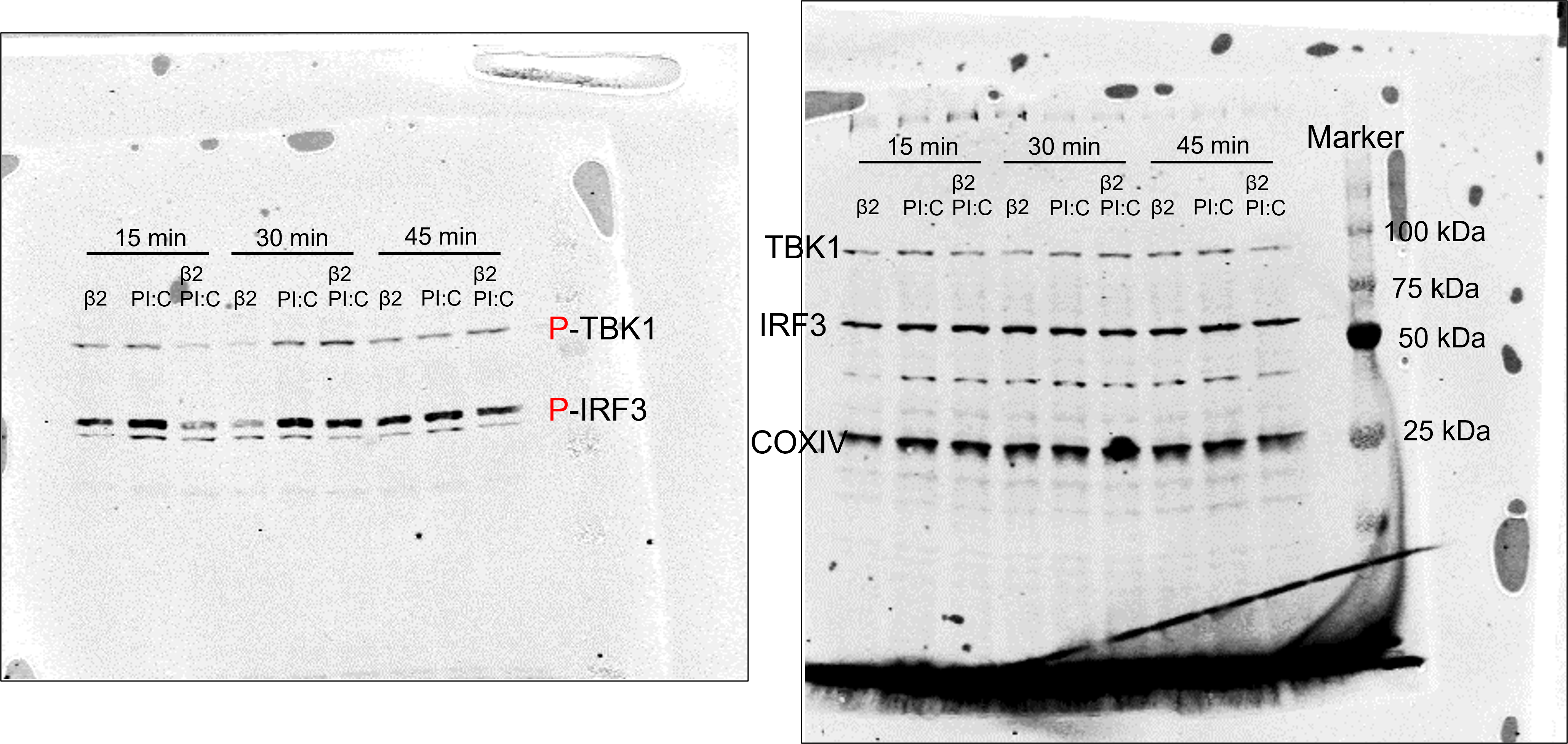

Supplement: Figure 3—source data 2. [file elife-66793-fig3-data2.tif]

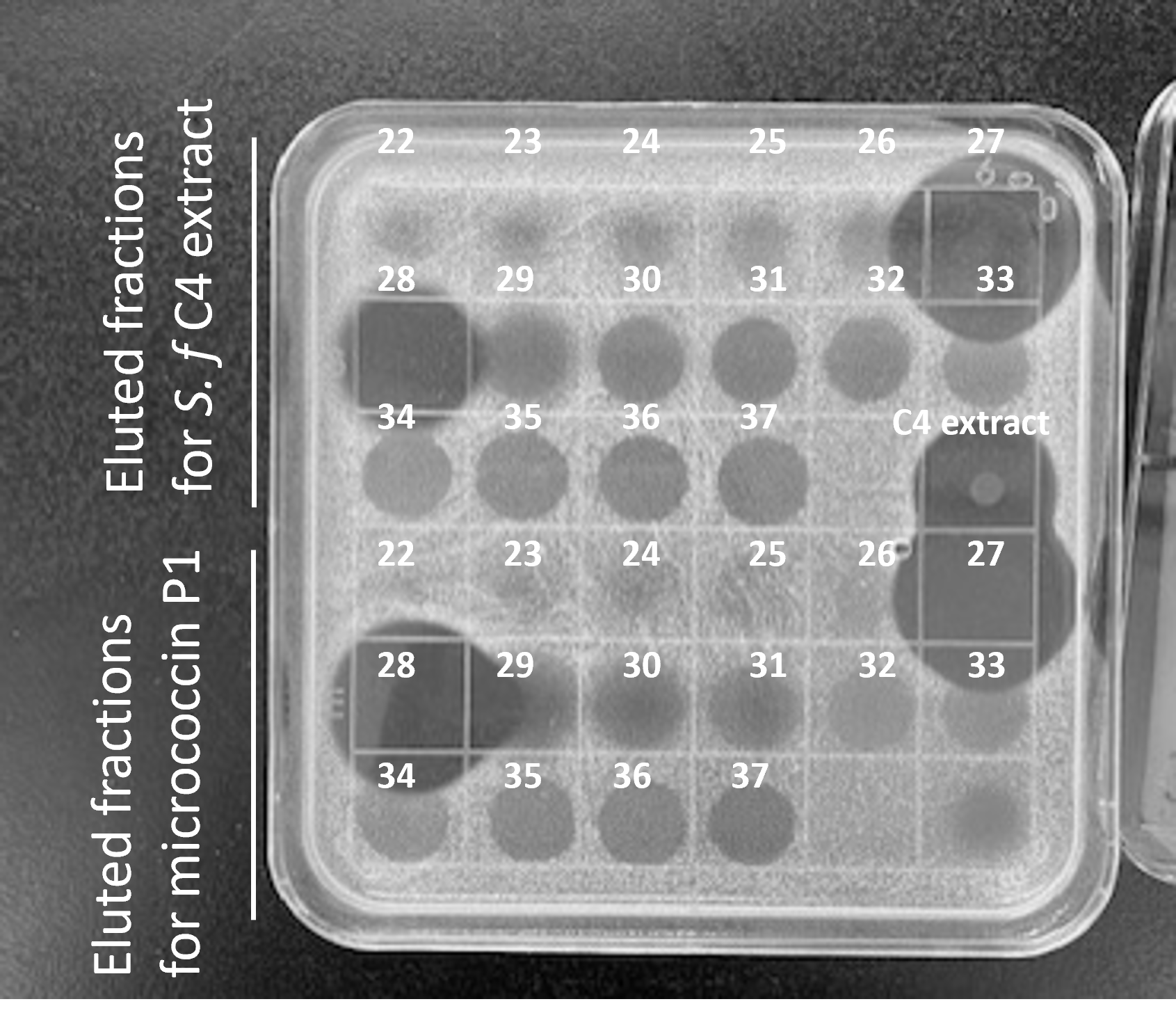

Supplement: Figure 4—source data 1. [file elife-66793-fig4-data1.tif]

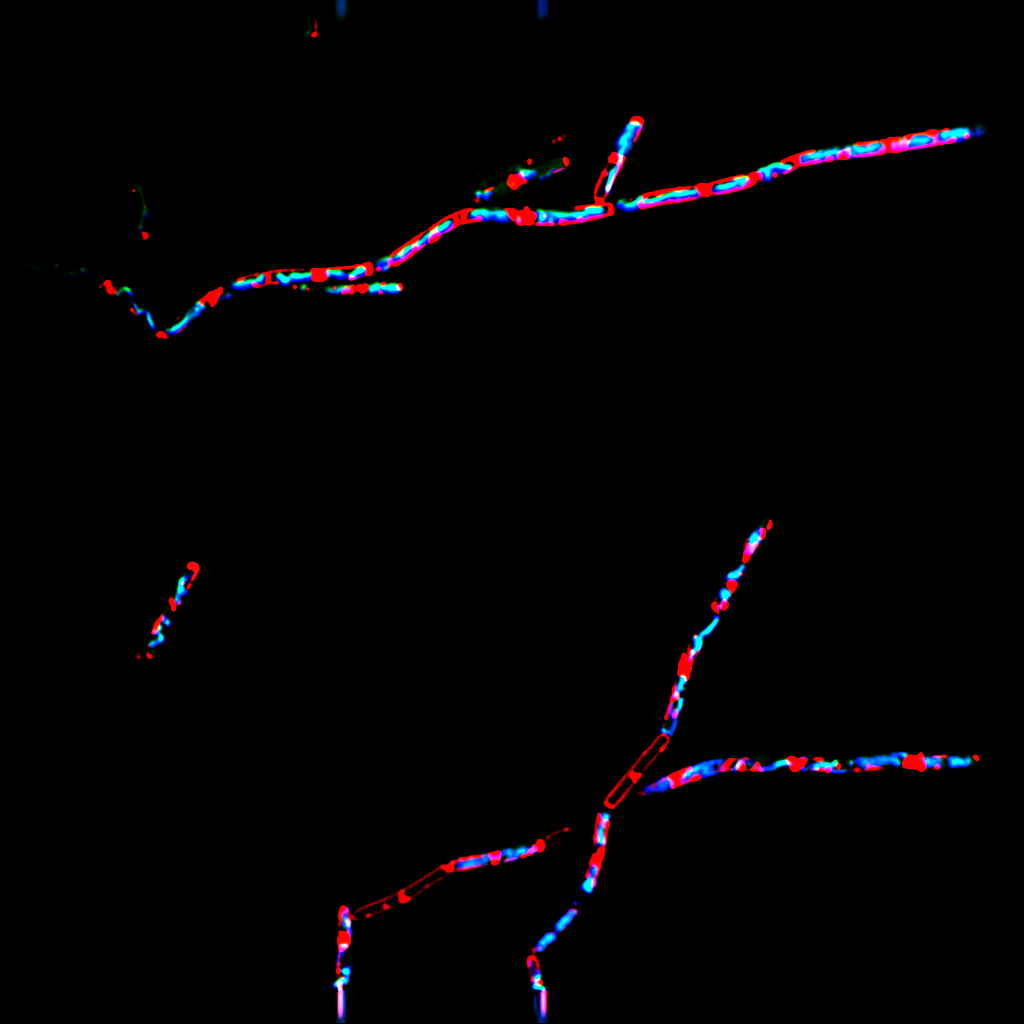

Supplement: Figure 5—source data 1. [file elife-66793-fig5-data1.zip › Figure 5 source data 1/PY79_TritonX.tif]

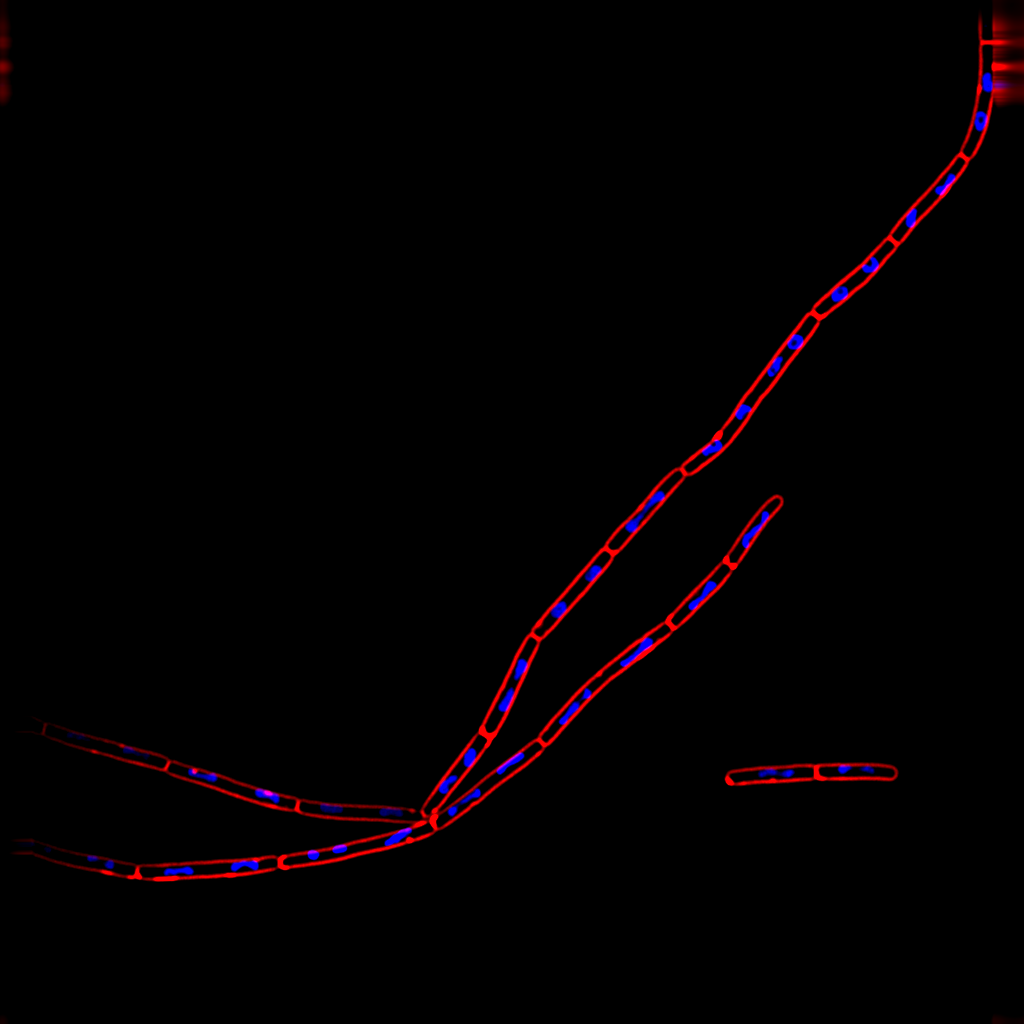

Supplement: Figure 5—source data 1. [file elife-66793-fig5-data1.zip › Figure 5 source data 1/PY79_Tetracycline.tif]

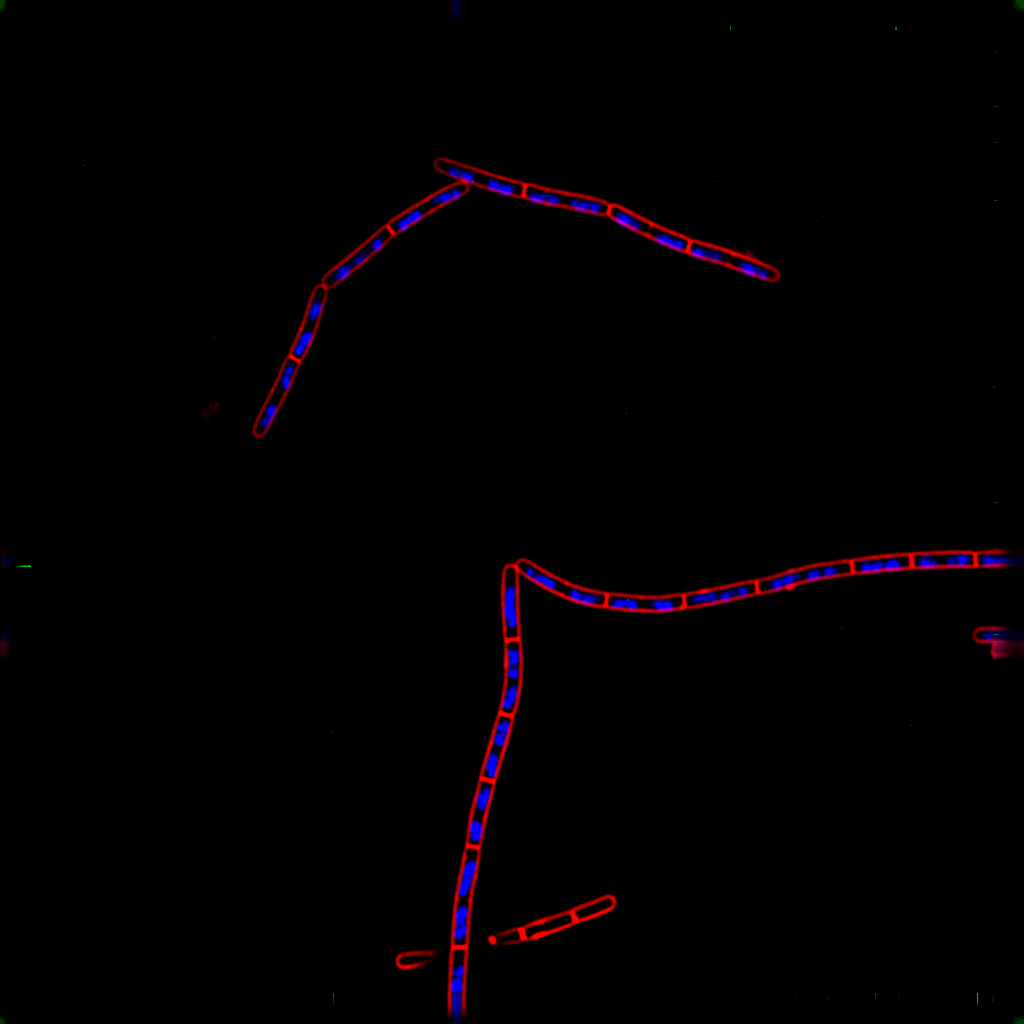

Supplement: Figure 5—source data 1. [file elife-66793-fig5-data1.zip › Figure 5 source data 1/PY79_PSMB2.tif]

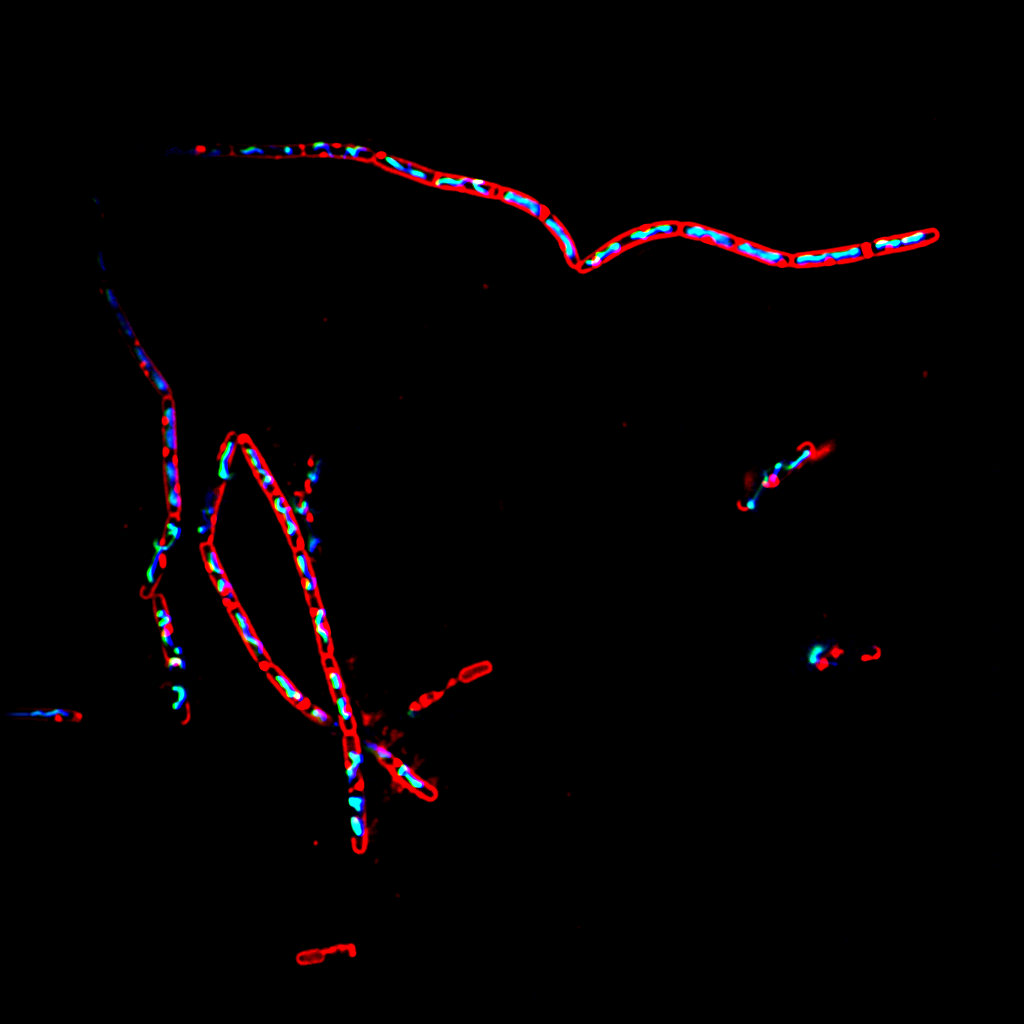

Supplement: Figure 5—source data 1. [file elife-66793-fig5-data1.zip › Figure 5 source data 1/PY79_Nisin.tif]

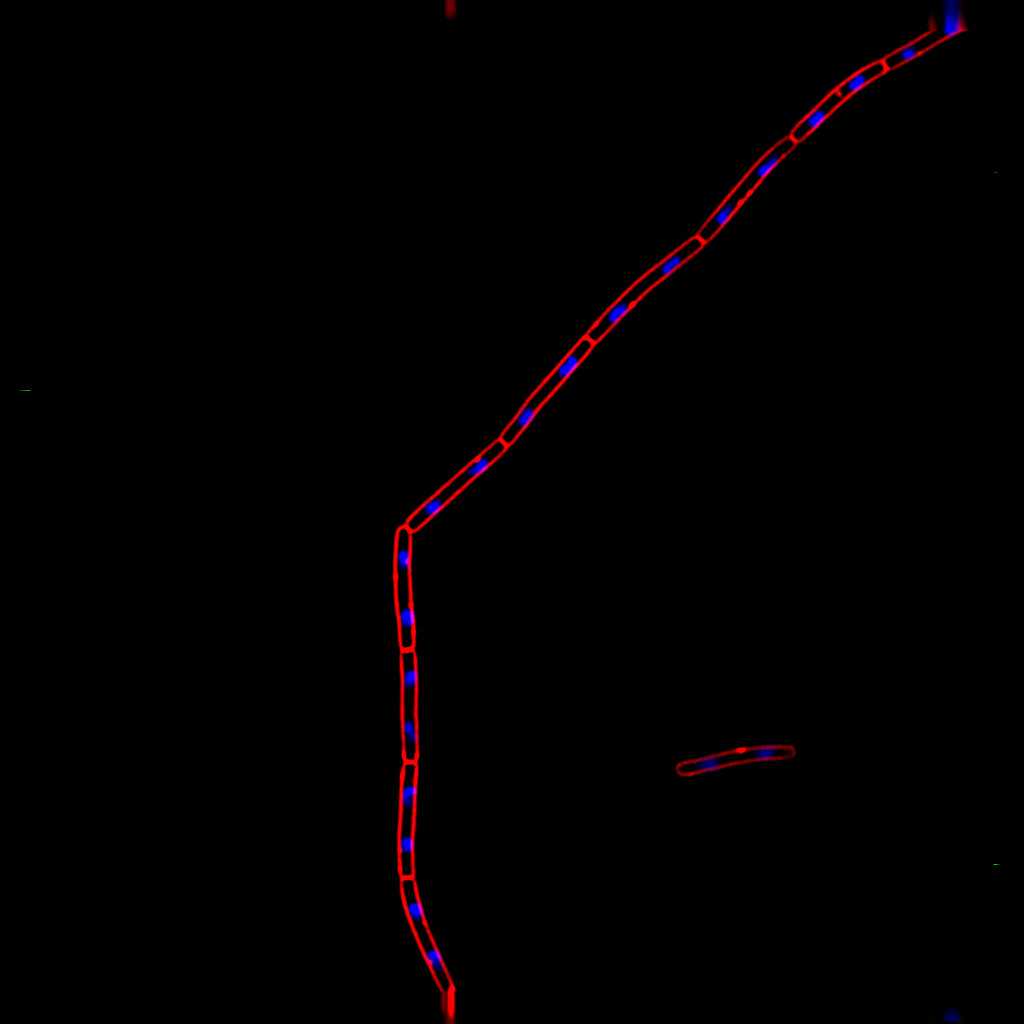

Supplement: Figure 5—source data 1. [file elife-66793-fig5-data1.zip › Figure 5 source data 1/PY79_Micrococcin.tif]

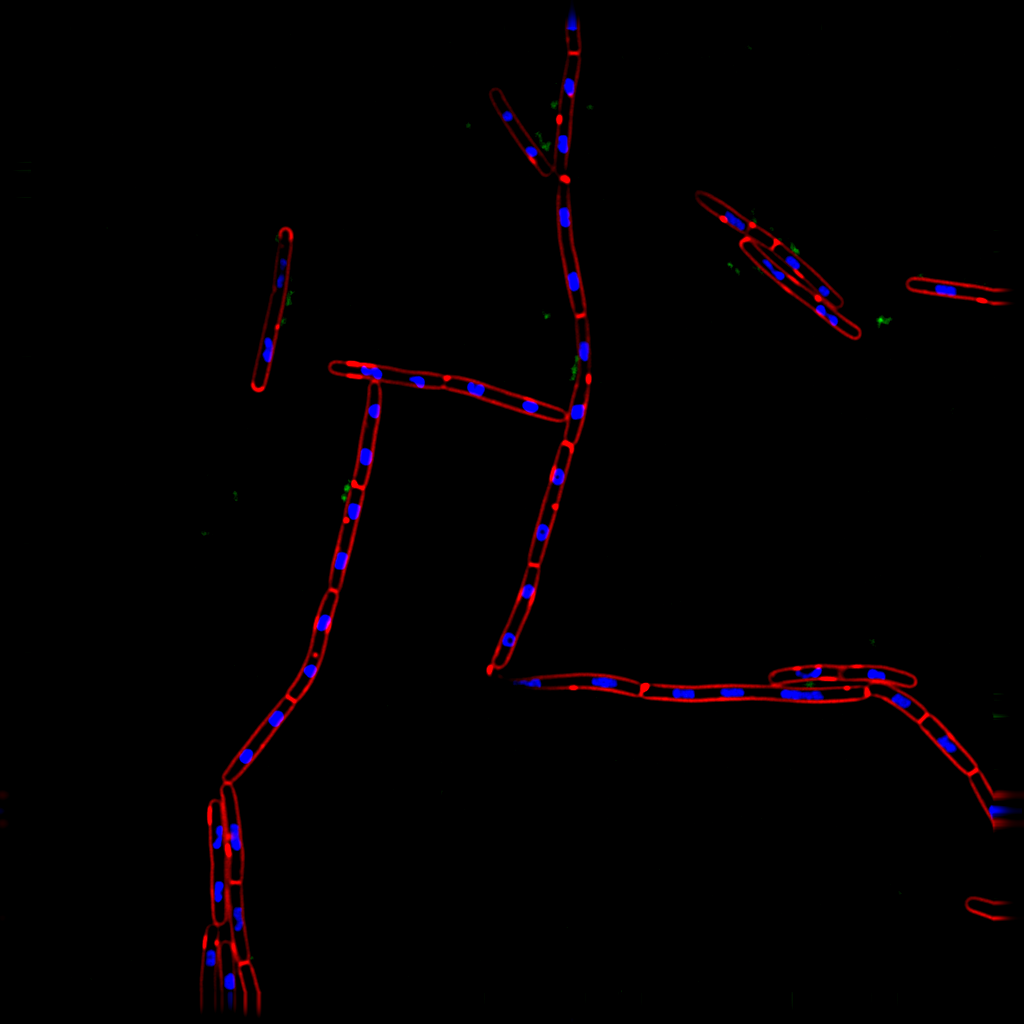

Supplement: Figure 5—source data 1. [file elife-66793-fig5-data1.zip › Figure 5 source data 1/PY79_Extract.tif]

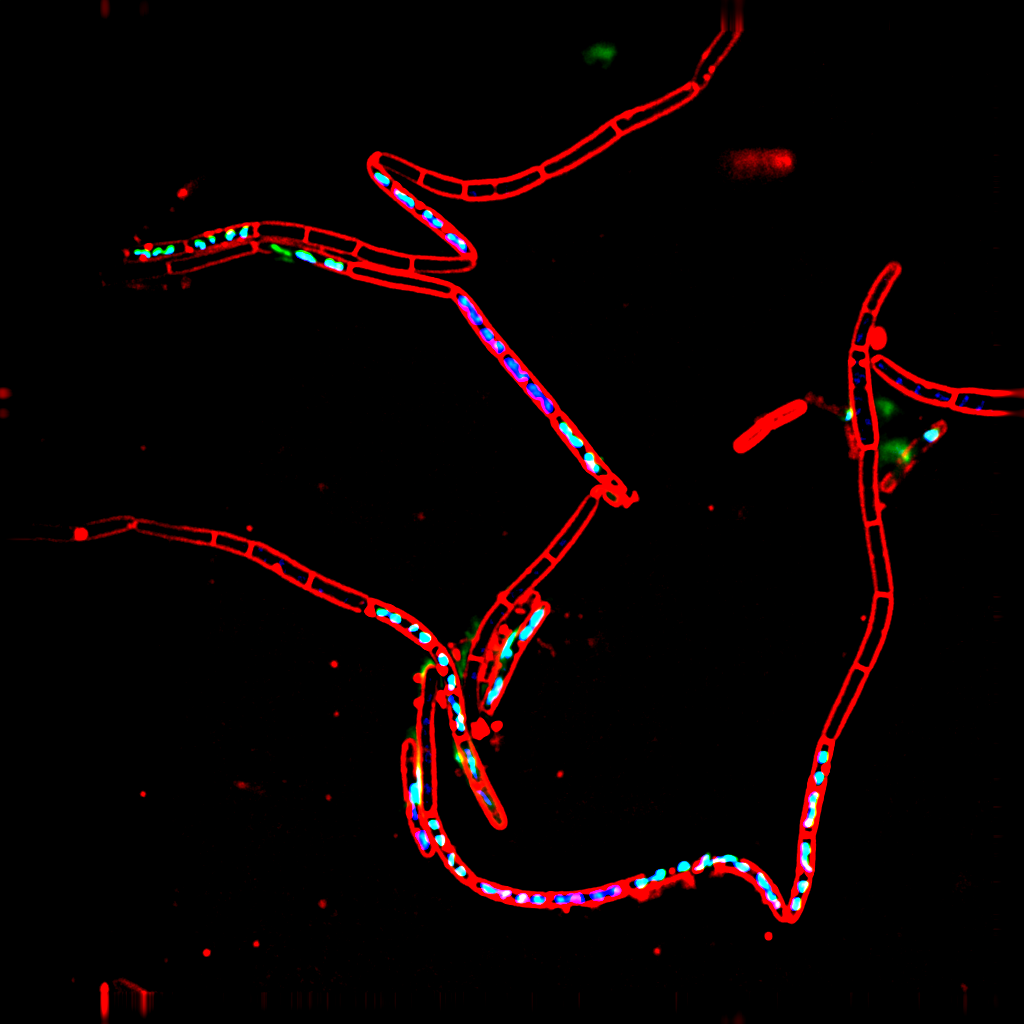

Supplement: Figure 5—source data 1. [file elife-66793-fig5-data1.zip › Figure 5 source data 1/PY79_cycloserine.tif]

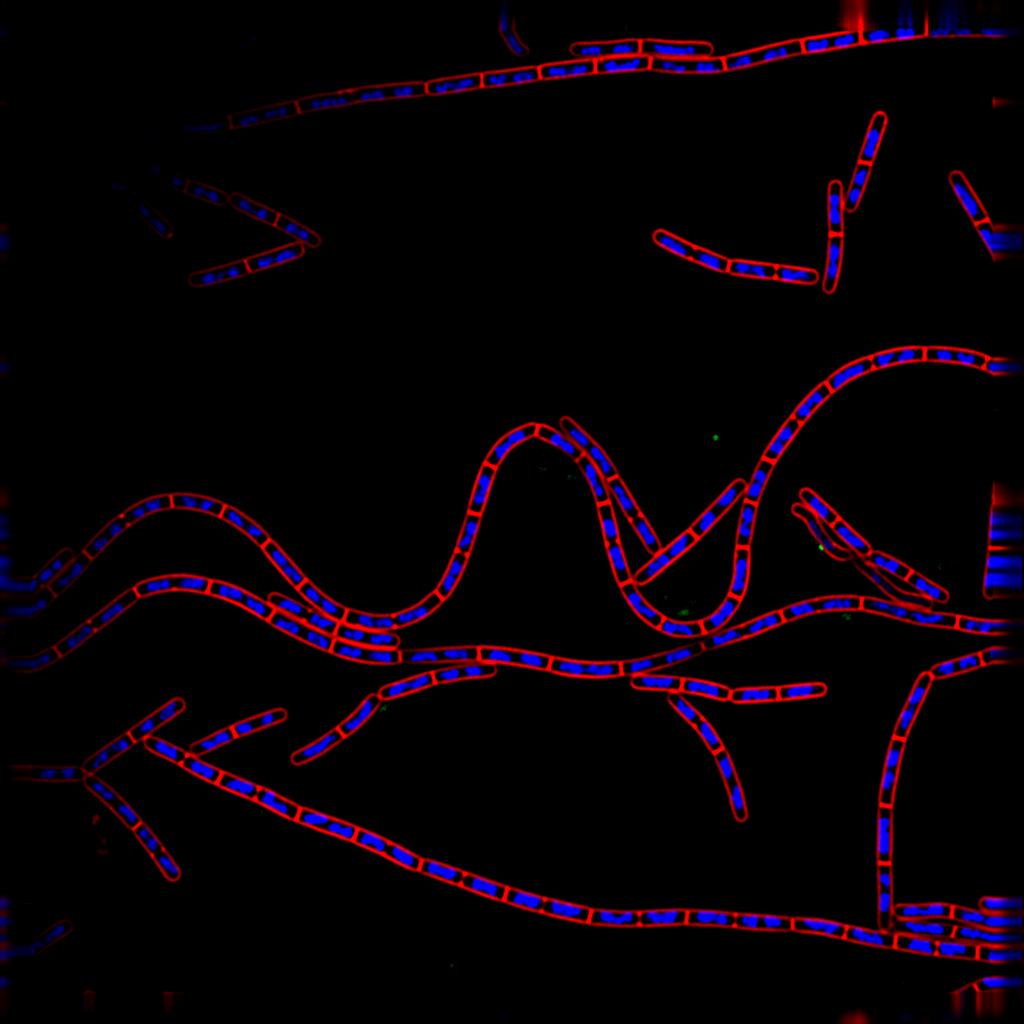

Supplement: Figure 5—source data 1. [file elife-66793-fig5-data1.zip › Figure 5 source data 1/PY79_CNTRL.tif]

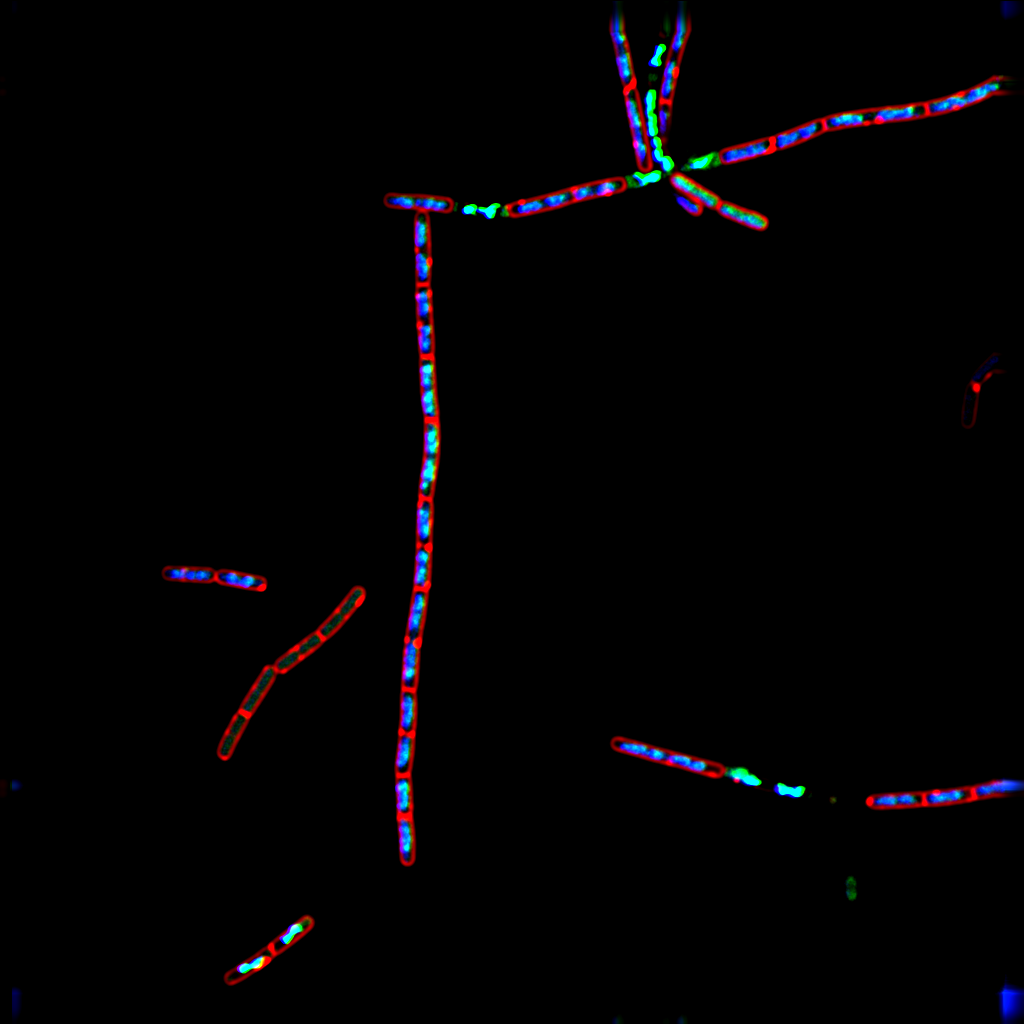

Supplement: Figure 5—source data 1. [file elife-66793-fig5-data1.zip › Figure 5 source data 1/PY79_CCCP.tif]

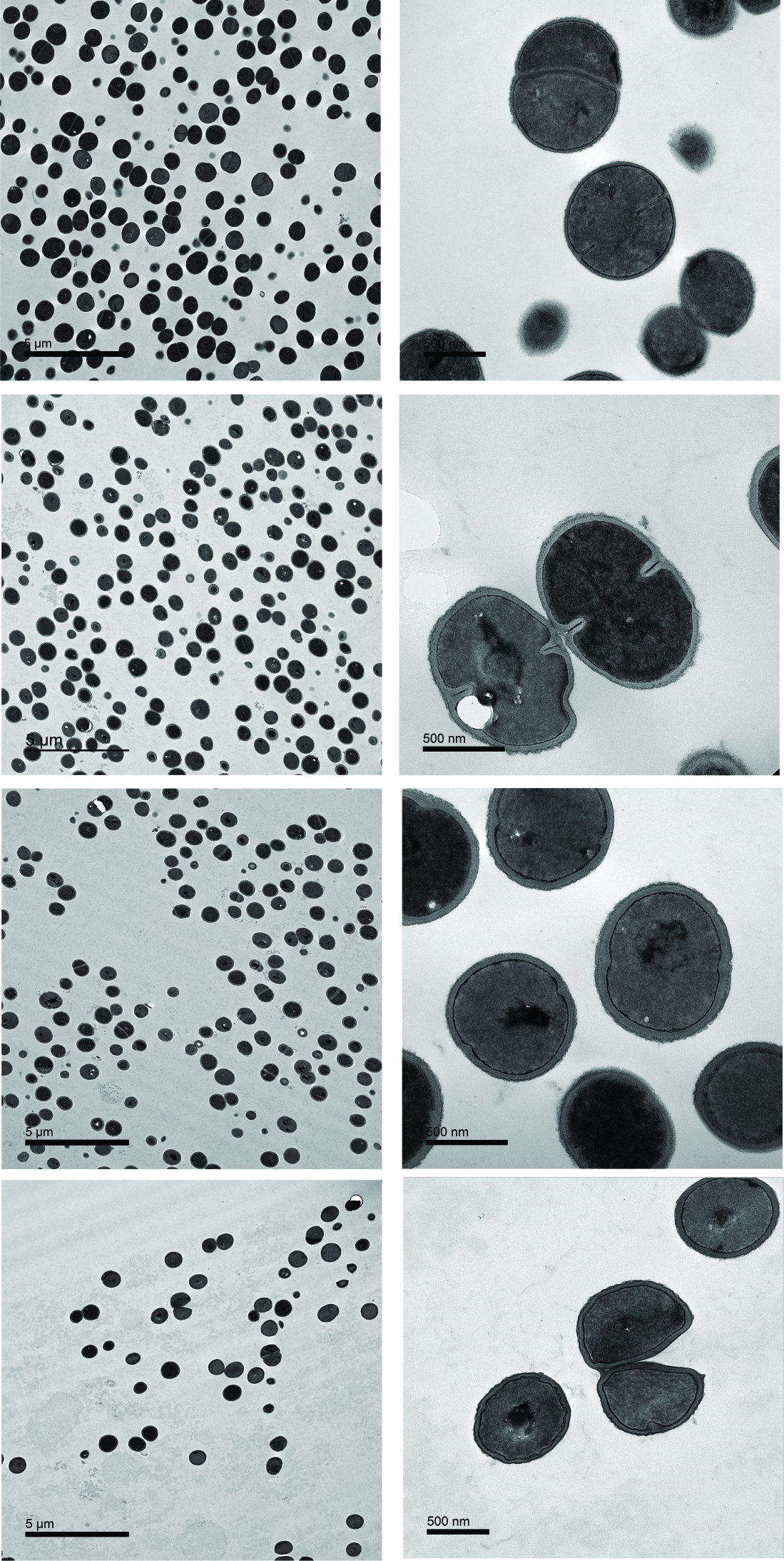

Supplement: Figure 5—source data 2. [file elife-66793-fig5-data2.tif]
